# Nitrogen-Assisted Foam Injection Molding of Polylactide with Carbon Fibers and PTFE Nanofibrils

**DOI:** 10.1021/acs.iecr.6c03418

**Published:** 2026-09-17

**Authors:** Silla George Raju, Valentina Volpe, Rita Salomone, Hanieh Kargarzadeh, Roberto Pantani, Andrzej Galeski

**Affiliations:** † BioMedChem Doctoral School of University of Lodz and Lodz Institute of the Polish Academy of Sciences, 21/23 Matejki Street, 90-237 Lodz, Poland; ‡ Centre of Molecular and Macromolecular Studies, 86897 Polish Academy of Sciences, Sienkiewicza 112, 90-363 Lodz, Poland; § Department of Industrial Engineering, University of Salerno, Via Giovanni Paolo II 132, Fisciano 84084, Salerno, Italy

## Abstract

Polylactide (PLA)-based foams are viable sustainable alternatives to traditional petroleum-derived foams owing to their intrinsic biodegradability, superior biocompatibility, light weight, and high specific strength, with potential applications in packaging, insulation, and biomedical fields. However, their inherently low melt strength and slow crystallization kinetics hinder the development of high-performance PLA foams. A reinforcement strategy combining in situ fibrillated polytetrafluoroethylene (PTFE) and carbon fibers (CFs) is applied to enhance the morphological, rheological, thermal, mechanical, and foaming behavior of PLA foams fabricated using nitrogen-assisted foam injection molding (FIM). The combined PTFE/CF reinforcement resulted in uniform cell nucleation and a refined microcellular foam structure. Rheological analysis showed a higher complex viscosity and storage modulus, indicating the formation of a percolated fibrillar network. Notably, extensional viscosity measurements showed that the incorporation of only 0.5 wt % CF induced pronounced strong strain hardening, leading to improved melt strength and foamability. The foam density of composites lowered 15% compared with pure PLA foam. The compressive strength and flexural stiffness improved significantly as compared to pure PLA foams by more than 11% and 20.5%, respectively. Furthermore, the thermal conductivity of the composite foam decreased by 21% with the addition of carbon fiber (CF), indicating improved thermal insulation. These findings demonstrate a promising strategy for developing PLA-based nanocomposite foams for lightweight structural and thermal-insulation applications.

## Graphical abstract

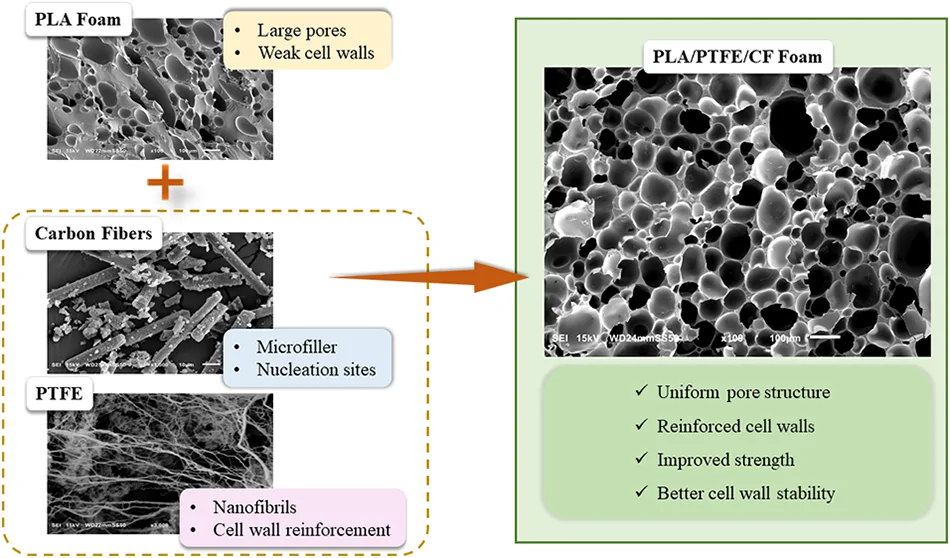

## 1 Introduction

Polylactide (PLA), an aliphatic polyester synthesized either by polymerization of D-lactic acid and L-lactic acid monomers^1^ from renewable resources such as starch and corn,^2,3^ stands out as a promising biodegradable and biocompatible thermoplastic polyester as an alternative to traditional petroleum-derived polymers. PLA possessing a low D-lactic acid content (less than 12%) is more suitable for foaming, which can result in higher crystallinity at elevated temperatures.^4^ As the D-lactic acid percentage increases, the crystallization kinetics become slower.^5^ It was reported that PLA with low D-lactic acid content produced a more uniform foam structure,^6^ with potential applications in construction, packaging, heat and sound insulation, and biomedical fields.^7,8^ However, low melt strength aligned with slow crystallization kinetics of PLA restricts its foamability,^9–11^ resulting in uncontrolled cell size and rupture.^12^

To address these issues, PLA has been modified using chain extenders, copolymerization, crosslinking, nanofiller addition, and blending.^13–16^ Among nanofillers, nucleating agents such as talc, clay, carbon fibrils, and cellulose fibrils have been reported to enhance this effect.^17,18^ Carbon fibers (CFs), in particular, are of interest due to their ability to improve the thermal insulation performance of foams,^19^ as well as increase the modulus and strength of the matrix.^20^ All-polymer nanofibrillated composite foams are another state-of-the-art technique to tackle the issue. It is based on forming distinctive fibrillar structures through a selective interface between PLA and the dispersed phase.^17,21–23^ The presence of a network of PTFE nanofibrils during blending, formed by a proper shearing in an intermeshing twin-screw extruder, has been shown to significantly enhance both the rheological behavior and foamability of PLA.^17,24–26^ The in situ fibrillation of PTFE results from its low chain entanglement density and the ease of plastic deformation of PTFE crystals. The transfer of shear stress from the molten matrix onto PTFE grains depends on the viscosity and interfacial tension between solid PTFE powder and molten polymer.^27^ PTFE crystals can be deformed by crystallographic chain slip under the shear stress of 2.5 MPa at room temperature, and it is lower than a single MPa at 200 °C. The other slip, the transverse slip, requires a significantly higher slip of 15 MPa at room temperature.^28^ We demonstrated^29^ that a network of PTFE nanofibers substantially increases the melt strength of thermoplastic polymers.^26,27,30–33^ Nanofibrils of PTFE exhibit strong nucleation of crystallization activity for several polymers, including PLA. That fact is especially relevant for the stabilization of PLA foams through crystallization.

The alliance of carbonaceous fillers such as carbon nanotubes (CNTs) with the PTFE nanofibrillar network provided additional centers of that network.^34^ However, the effect would be more pronounced if CNTs were stiffer and had a lower tendency to become tangled and clump together. This was our incentive to choose CFs for the present research and not carbon nanotubes as a second additive to PTFE. However, achieving a uniform dispersion of nanoscale additives within the polymer remains a critical challenge. Optimization of processing parameters prior to foaming is therefore essential to ensure homogeneous cell nucleation, a uniform cell size distribution, and high cell density. Upon optimization, these nanoscale reinforcements may align flat along cell walls as fibers, imparting additional strength and stability to the foam structure.^33,35^

Foam injection molding (FIM) represents a promising technique with high efficiency, low cost, dimensional stability, short cycle times, and reduced material consumption, suitable for foaming biopolymers with narrow thermal processing windows and limited thermal stability.^36,37^ Extensive studies on FIM have highlighted the effect of nanoparticles in improving the foamability of PLA by enhancing melt strength and increasing cell nucleation efficiency.^37,38^ Bing et al. reported that the incorporation of a nucleating agent during the microcellular injection molding process resulted in a final crystallinity of approximately 55.7% for the PLA foam, occurring through decreased apparent and local crystallization activation energy compared to neat foamed PLA.^39,40^ Moreover, the addition of CFs increased the melt viscosity and elasticity of composites.^41^ It was found that a self-assembled fiber framework, with the further induction of PLLA crystallization during cooling foaming, can enhance the melt strength of the PLA for foaming.^42^ A novel strategy was developed combining in situ fibrillation of PTFE and mold-opening microcellular injection molding (MOMIM) to fabricate lightweight and tough PLLA/PTFE foams.^43^ The improved melt strength, due to the accelerated PLA crystallization from PTFE nanofibrils, significantly improved the cell density of the resulting PLA foams.^44^ Despite the reported studies on the individual CFs and PTFE fillers, to the best of our knowledge, there are no specific studies on the FIM of PLA reinforced with hybrid in situ-generated PTFE and carbon fibers.

The work presents the studies of the structure and properties of PLA foams and foams of PLA nanocomposites with carbon fibers and PTFE nanofibers generated during nitrogen-assisted FIM compounding. Foaming was achieved by injection molding aided by supercritical nitrogen at a pressure above 34 atm.^45^ The composites, with improved melt viscoelasticity and foamability, were characterized using many techniques, including DSC, rheology, SEM, light microscopy, X-ray tomography, mechanical testing, and thermal conductivity. In the view of all of these experimental results, it became evident that the addition of carbon fibers (micrometer-scale filler) facilitates nucleation of more bubbles, while PTFE nanofibers reinforce cell walls. The synergy of micrometer carbon fibers and nanometer PTFE fibrils improved cell nucleation and led to a refined cell morphology, as evidenced by X-ray tomography.

## 2 Experimental Section

### 2.1 Materials

Commercial PLA pellets (2003D grade, density: 1.24 g/cm^3^, d-isomer content: 4.3%, melt flow rate: 6 g/10 min at 210 °C/2.16 kg load) were purchased from NatureWorks LLC, USA. PTFE powder flakes with an average particle size of 28 μm and a melting point of 345.7 °C were supplied by DuPont. Carbon fibers (purity > 99.9%) were purchased from Nanoshel LLC, Wilmington (USA). Nitrogen gas was used as the physical foaming agent for foam injection molding.

### 2.2 Preparation of Samples

#### 2.2.1 Preparation of Composites

All materials were dried in a convection oven at 70 °C for 24 h prior to processing to avoid hydrolytic degradation during melt processing. Composites of PLA/PTFE/CF were prepared with an optimal fixed PTFE content of 1 wt % and varying CF loadings of 0.5, 1, 3, and 5 wt %. The formulations and corresponding sample designations are outlined in Table 1. Melt compounding was employed using a co-rotating twin-screw extruder (2 × 20/40D EHP, Zamak Mercator, Poland) at a temperature profile maintained at 170, 175, 175, 180, 180, 185, 180, 175, and 175 °C from the hopper to the die, with a fixed screw speed of 50 rpm. The extrudates were cooled and pelletized for further characterization and processing.

**Table 1:** Formulation Table of Prepared PLA Composites

samples	Pure PLA	PLA/PTFE/CF0.5	PLA/PTFE/CF1	PLA/PTFE/CF3	PLA/PTFE/CF5
CF (wt %)	0	0.5	1	3	5
PTFE (wt %)	0	1	1	1	1

#### 2.2.2 Preparation of Foams

A 70-ton Negri-Bossi injection molding machine with a screw diameter of 25 mm and an *L*/*D* ratio of 22 was employed to produce foam specimens. The injection molding unit was fitted with a volumetric pump to regulate the gas dosage injected during the batching phase. A screw incorporating a mixing section was used to facilitate the gas dissolution within the polymer melt. To prevent premature foaming, a nozzle equipped with a needle valve was utilized, while a hot runner system was adopted to prevent solidification within the channel. Nitrogen in its supercritical state served as the physical foaming agent for the FIM process. The processing conditions used are listed in Table S1. The molded specimen (Figure 1) is composed of two distinct regions: a neck and a base. The “neck” exhibits a greater thickness along the cross-flow direction, whereas the “base” is distinguished by radial shear flow, resulting from the injection point strategically positioned at the bottom center of the base. The particular geometry of the sample enables to observe different characteristics of foam injection molding.^37^

**Figure 1:**
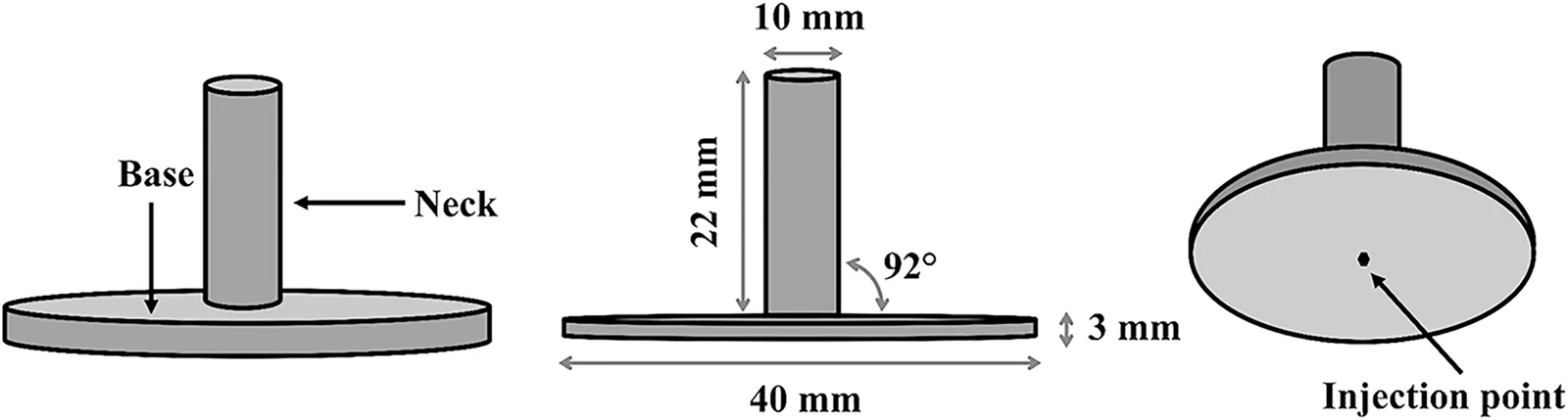
Schematic illustration of the molded specimen geometry and its dimensions.

The processing parameters (Table S1) were chosen to achieve optimal molded specimens. Specifically, the shot volume (dosage) was optimized to ensure total filling of the mold cavity and formation of a complete sample, with consideration of expansion in the case of foamed samples. Accordingly, the injected shot volume differed between foamed and compact specimens, with dosage volumes of 15 mm and 20 mm, respectively. Compact samples were fabricated by using conventional injection molding techniques to facilitate subsequent comparative characterization studies with foamed counterparts.

### 2.3 Characterization

#### 2.3.1 Scanning Electron Microscopy (SEM)

Scanning electron microscopy (JEOL JSM-5500 LV) was used to examine the cryofracture surface of composites and foams at 15 kV. The fractured surfaces were sputtered with a 20 nm gold layer using an ion sputter coater (JEOL JFC-1200). To have insight into the PTFE fibrillar dispersion and their network structure in the PLA matrix, the composite sample was etched using N,N-dimethylformamide (DMF) solvent at 60 °C for 1 h, as per.^17^

#### 2.3.2 Rheological Measurements

Rheological measurements were carried out using the Haake Mars—Modular Advanced Rheometer System (Thermo Scientific) with a parallel plate configuration and an imposed gap of 1 mm. The frequency sweep tests were performed at 180 °C, over a frequency range of 0.1–100 rad/s. The experiments were performed using pelletized pure PLA and all composites prior to foam injection molding. Uniaxial extension tests of selected molten samples were performed using an extensional viscosity fixture (EVF, TA Instruments) attached to the ARES rheometer (TA Instruments). The 18 × 10 × 0.7 mm^3^ rectangular samples, prepared by hot-compression molding at 180 °C, were uniaxially extended at Hencky strain rate, ε̇, of 0.2 s^–1^. The extensional viscosity as a function of time was measured five times for each sample.

#### 2.3.3 Differential Scanning Calorimetry (DSC)

Differential scanning calorimetry (Q20, TA Instruments) was used to analyze the thermal characteristics of the samples (6–7 mg) at heating and cooling rates of 10 °C/min under nitrogen flow. The crystallinity (*χ*_c_) of PLA was calculated using eq 1

1 $$\chi_{c}\;\left(\%\right)=\left(\frac{\Delta H_{m}-\Delta H_{\mathrm{cc}}}{\Delta H_{m}^{0}\times \left(1-w_{t}\right)}\right)$$

where Δ*H*_cc_ refers to the cold crystallization enthalpy, Δ*H*_m_^0^ is the melting enthalpy of 100% crystalline PLA, and *w*_t_ is the filler weight fraction in the PLA/PTFE/CF composite.

#### 2.3.4 X-ray Tomography

Computer-processed X-ray tomography analysis was conducted using CT Lab HX130 (Rigaku) operating at a voltage of 90 kV and a current of 60 μA to obtain the tomographic images, or slices, of the specific parts of the injection-molded samples. Scanning was performed without the use of any filters; the voxel size was 17.6 μm.

#### 2.3.5 Foam Structure Analysis

The density measurements were performed according to Archimedes’ principle by weighing a minimum of five samples, base and neck, while immersed in water at 25 °C. The density reduction percentage, *R* (%) was determined using eq 2

2 $$R\;\left(\%\right)=\left(\frac{\rho_{0}-\rho_{f}}{\rho_{0}}\right)\times 100$$

where *ρ*_0_ and *ρ*_f_ represent the density of the compact and foamed samples, respectively.

The foam expansion ratio (ϕ) of PLA foams was measured using eq 3

3 $$\phi =\frac{\rho_{0}}{\rho_{f}}$$

The cell concentration (*N*_0_) per unit volume (cm^3^) of PLA foams was computed by eq 4

4 $$N_{0}={\left(\frac{n}{A}\right)}^{3/2}\left(\phi \right)$$

where *n* represents the cell number and *A* represents the area of the SEM micrograph (in cm^2^). The cell concentration values were quantified from five SEM micrographs, with a minimum of 75 cells analyzed per image acquired from at least two independent fracture surfaces.

#### 2.3.6 Compression Measurement

The compression behavior of the foam composites was studied using a universal testing machine (ATSFAAR TC1000). The cylindrical part of the sample (neck), measuring 22 × 10 mm (length × diameter), was subjected to axial compression using a 10 kN load cell at a speed of 4 mm/min. A minimum of five specimens per set of condition parameters were tested.

#### 2.3.7 Thermal Insulation Properties

Thermal conductivity tests were analyzed using a purpose-built apparatus consisting of two foil heat flux analyzers coupled with two thermal sensors (Hukseflux FHFo1, Delft, Hol), two thermocouple-to-analog converters (OMEGA Engineering TAC80B–K, Norwalk, USA), a USB data-capturing system (National Instrument USB6210, Austin, USA), two signal amplifiers (Analog Devices 5B40, Wilmington, USA) installed onto a motherboard, and a power supply (Agilent E3630A, Santa Clara, USA). A LabView code was created in order to obtain the values of temperature, heat flow, and thermal conductivity signals. The experiments were conducted at 60 °C for a sample possessing a thickness of ∼5 mm.

## 3 Results and Discussion

### 3.1 Morphological Properties of Composites

CFs are found as solid, individually separated short-fiber morphology with an average diameter of 8 μm (Figure S1). PTFE exhibited irregularly shaped flake-like particles with varying diameters (Figure 3a). PLA shows a flat and smooth fractured surface (Figure S2), corresponding to its brittle character. Conversely, the addition of fillers into the PLA matrix developed a nonuniform and rough surface due to strengthening and toughening of the matrix (Figure 2a–d).^46,47^ The transition of PTFE flakes into nanofibrillar morphology when processed under high shear^26^ is observed for all composites with thin fibrils of PTFE with an average diameter of ≤1 μm visible on the surface. This results from the low yield strength and plastic deformation during PTFE blending with the PLA matrix.^29,48^ The incorporation of PTFE up to 1 wt % was found optimal with effective dispersion during melt compounding.^31^ Figure 3b confirms the formation of a nanofibrillated PTFE network structure upon high shear force experienced during melt mixing in the extruder and injection molding.

**Figure 2:**
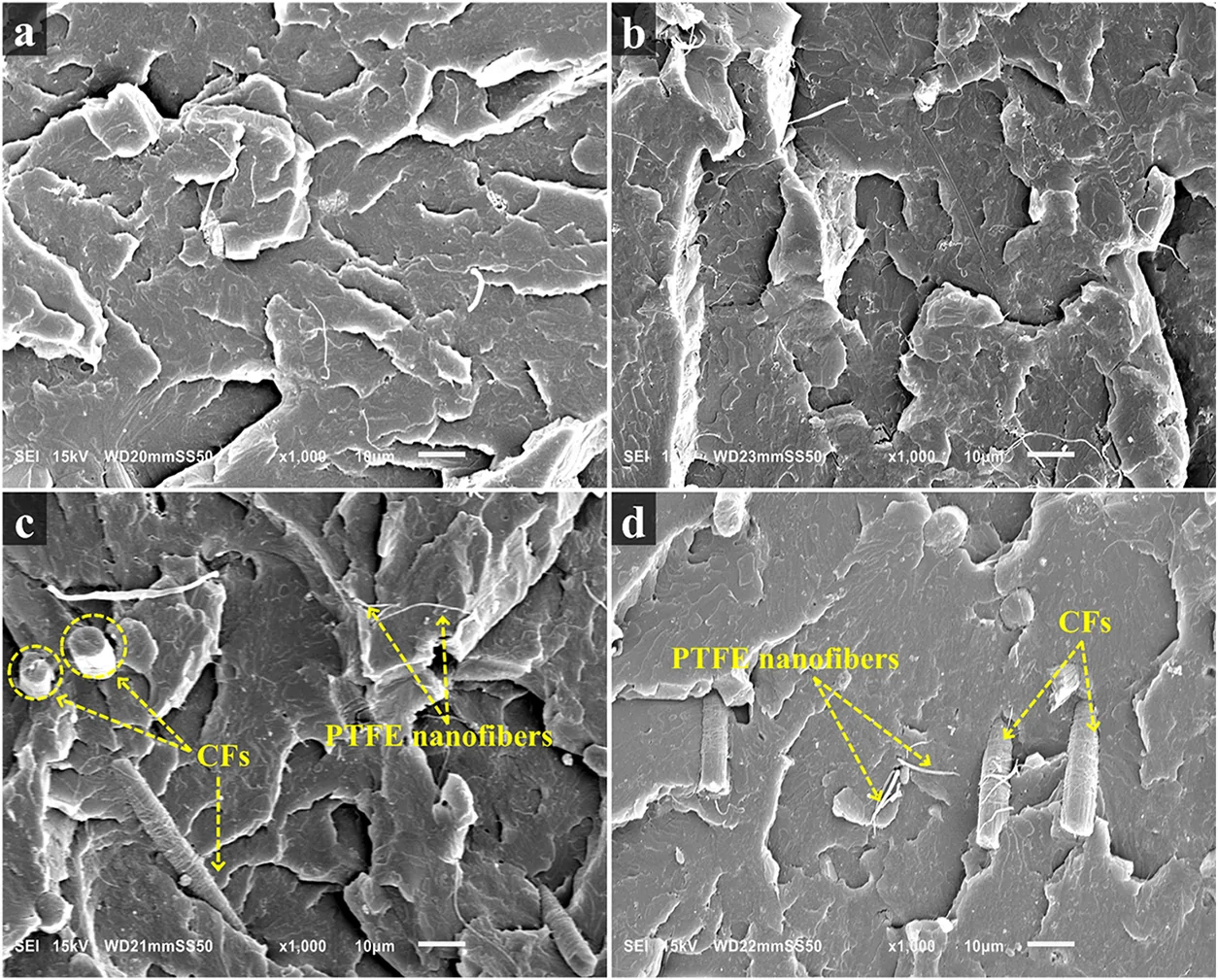
SEM micrographs of composites (a) PLA/PTFE/CF0.5, (b) PLA/PTFE/CF1, (c) PLA/PTFE/CF3, and (d) PLA/PTFE/CF5.

**Figure 3:**
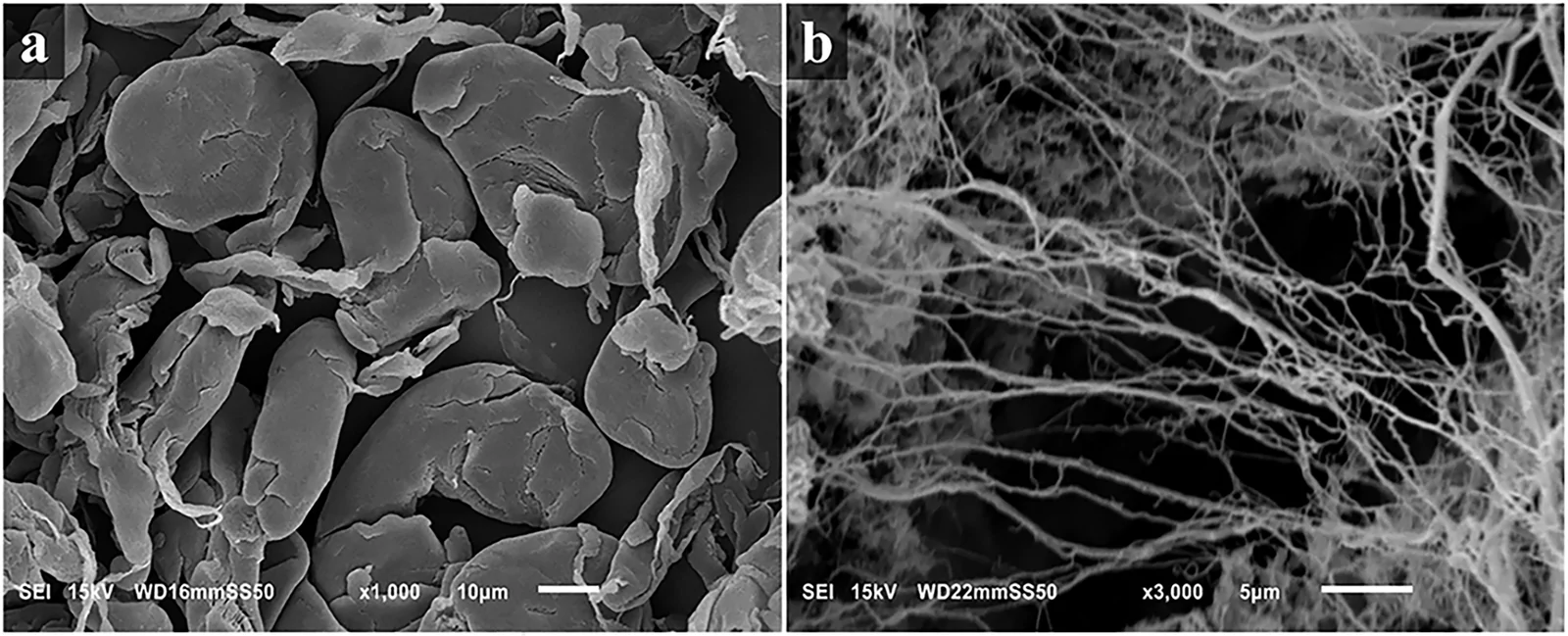
SEM micrographs of (a) PTFE particles and (b) network of PTFE nanofibrils in PLA exposed by solvent etching.

CFs were rarely visible in Figure 2a,b due to the lower concentration of filler inside the matrix. At 3 and 5 wt % of CFs, fiber-like structures were clearly visible protruding from the fractured surface (Figure 2c,d). However, no agglomeration of CFs was seen even at higher filler concentrations, indicating that CFs were effectively dispersed. The reason for this enhanced dispersion of CF is due to the PTFE fibrillation, which improves the melt viscosity of the material, resulting in uniform dispersion due to the high shear effect during the melt-compounding process inside the twin-screw extruder. A similar phenomenon was also reported by Huang et al. in polybutylene succinate (PBS), highlighting the effective dispersion of CNT in the PBS/CNT/PTFE composite due to the increased viscosity by the formation of PTFE fibrils.^24^

### 3.2 Rheological and Thermal Properties of the Composite

Figure 4 depicts the results of frequency sweep experiments, illustrating the dependencies of (a) storage modulus (*G*′) and (b) damping factor (tan δ) as functions of angular frequency (ω) for pure PLA and its composites at 180 °C. The *G*′ (Figure 4a) values of the composites were higher than those of pure PLA, which can be ascribed to restrained chain relaxation triggered by the occurrence of PLA-PTFE-CF, CF-CF, PTFE-PTFE, and PTFE-CF networks. A comparison of the linear viscoelastic response of PLA with PLA/PTFE/CF composites reveals a significant effect of fibrillar structures originating from CF and PTFE, especially at low frequencies. The higher *G*′ value of the composite relative to pure PLA indicates an enhancement in melt elasticity. Moreover, the emergence of an apparent plateau at low frequencies suggests the presence of percolated fibrillar networks within the molten PLA matrix. These networks act as microstructures capable of storing deformation energy over longer time scales, thereby significantly enhancing the elastic response. However, the G′ curves of the composites remain relatively close, indicating that the addition of fillers preserves the short-range dynamics of the PLA chains.

**Figure 4:**
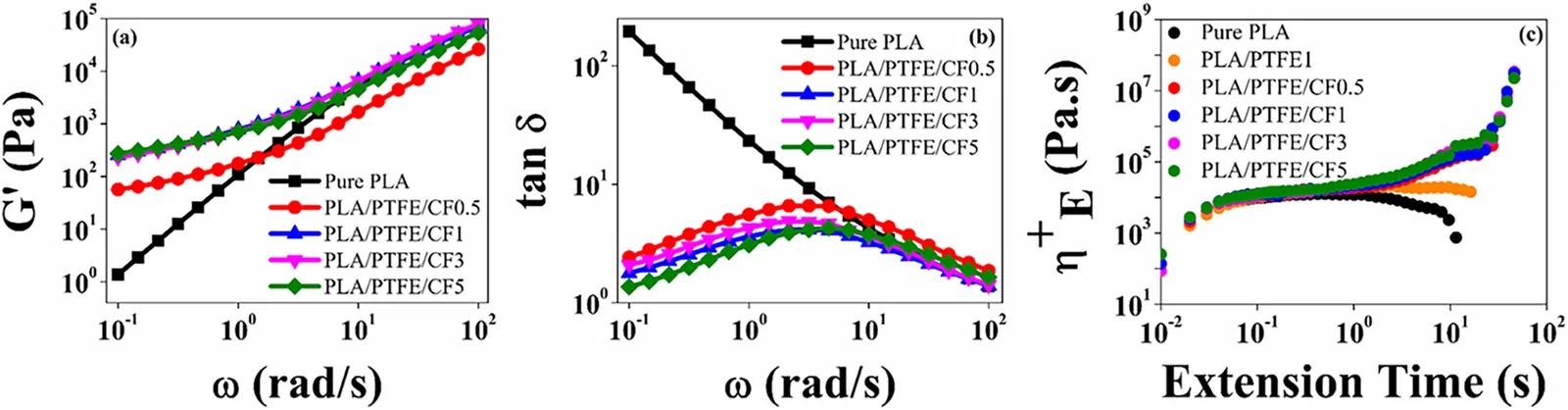
Rheological behavior of pure PLA and its composites: (a) storage modulus (*G*′), (b) tan δ as a function of angular frequency, and (c) elongational viscosity as a function of time at 180 °C.

The curve of tan δ plotted as a function of frequency in Figure 4b reveals a sharp decrease with increasing frequency for pure PLA. The addition of PTFE/CF fillers into the PLA matrix significantly improved the elastic response of the composites at low frequencies, attributable to the observed nanofibrillated networks of PTFE fibers within the PLA matrix.^49^ The suppression of tan δ is further enhanced by increasing the concentration of CF from 0.5 to 5 wt %. The increased tan δ value of composites at around 2 rad/s indicates a more plastic behavior region, arising from the breakdown of fibrillated networks under applied shear stress. Comparable trends have been reported for PBS/CNT/PTFE composites.^24^

Figure 4c shows the extensional viscosity, *η*_*E*_^+^, as a function of time, measured at 180 °C at the extension rate of 0.2 s^–1^ for pure PLA, PLA with an embedded network of 1 wt % of PTFE nanofibrils, and PLA/PTFE/CF hybrid nanocomposites. Pure PLA itself does not show any strain hardening during extensional viscosity measurements. Compounding of PLA with the addition of 1% of PTFE produced a network of PTFE nanofibers, which increases the viscous response of the composition; however, not above the level of strain hardening at 180 °C. The addition of a small amount of CF (0.5%) to the system drastically changes the response, evidencing a strong and significant strain hardening. The alliance of CF with the PTFE nanofibrillar network appears to be effective through the formation of additional centers of that network, which was based on our earlier work.^34^ Figure 5 presents representative SEM micrographs of the PLA/PTFE/CF5 composite after mild selective etching of the PLA matrix, revealing the residual PTFE nanofibrillar network anchored to the CF surface. At lower magnification (Figure 5a), an approximately 8 μm diameter CF protrudes from the fractured PLA fragments. Along its exposed surface, numerous curled PTFE fibrils extend into the voids created by PLA removal. These fibrils, a few hundred nanometers wide, appear to be attached to the CF surface rather than to the surrounding PLA debris. The higher-magnification image (Figure 5b) further reveals a continuous, interconnected PTFE nanofibrillar mesh, approximately 500-900 nm wide, surrounding the CF and forming a web-like structure between adjacent PLA/CF fragments. The contacts between PTFE fibrils and CF act as additional physical network junctions. This morphology is consistent with the rheological results, particularly the elongational viscosity, suggesting that network connectivity saturates once a sufficient number of well-dispersed CF nodes is present. Further CF addition beyond this threshold does not proportionally increase the number of effective network junctions.

**Figure 5:**
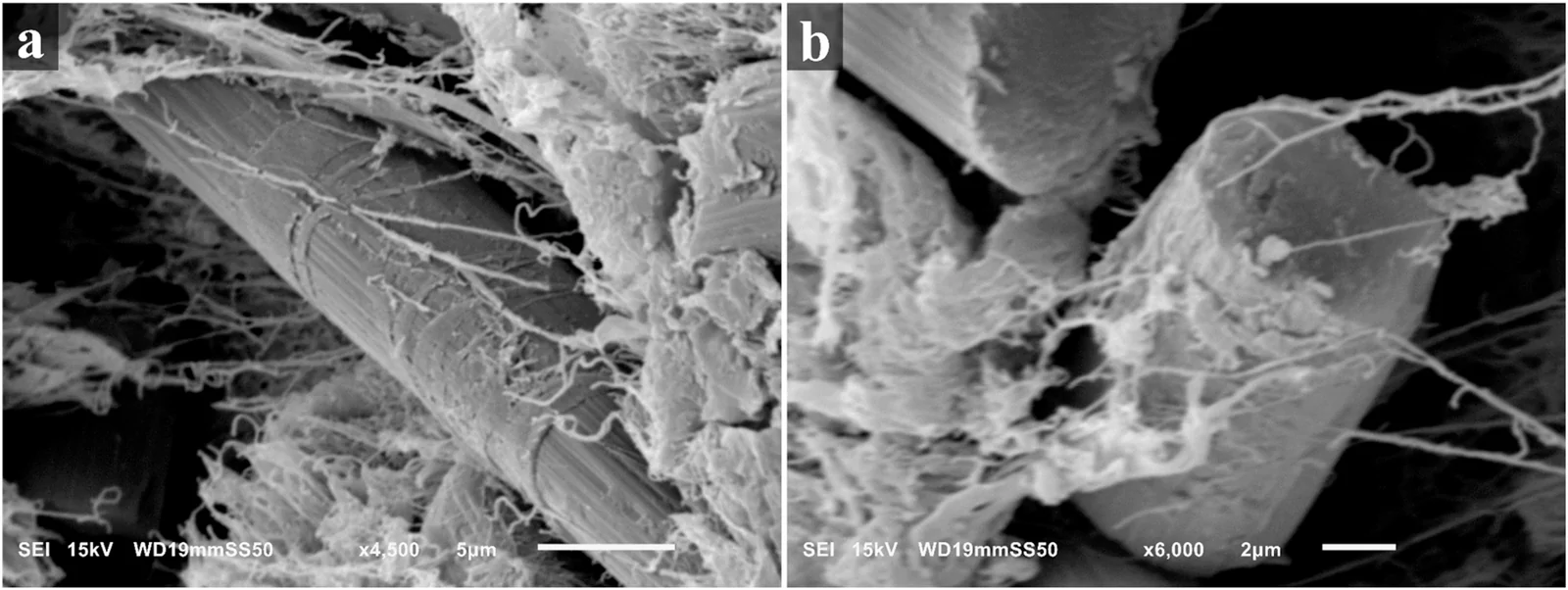
SEM micrographs of the PLA/PTFE/CF5 composite after mild selective etching of the PLA matrix, revealing the residual PTFE nanofibrillar network anchored to the CF surface. (a) 5 μm and (b) 2 μm.

As shown in Figure S4a, all composite samples, in contrast to pure PLA, display a broad and wide cold crystallization process. Notably, the addition of PTFE into PLA resulted in a more intense peak and shifted the cold crystallization peak to a slightly higher temperature (approx. by 5 °C) in contrast to pure PLA (Table S2). This occurrence could be associated with the nucleation ability of PTFE for PLA crystallization and with the restriction of movement in the molecular chains of PLA.^10^ The development of an entangled network between the polymer–filler and filler–filler has established a confinement effect on PLA chain dynamics and has constrained the growth of PLA spherulites, leading to a higher value of *T*_cc_.^10,50^ A gradual increase in *T*_cc_ was observed with increasing CF content, indicating the ability of CF to act as nucleating agents (Table S2). Similar behavior was also found and reported by Ludwiczak et al.^51^ for PLA foams in the presence of a chain extender. The shift in *T*_cc_ for composites to higher temperatures indicates the potential of the sample to possess crystallization under cooling as a result of the presence of heterogeneous nucleation surfaces.^52^

The melting temperatures of neat PLA and the composites were similar, whereas filler addition increased the melting enthalpy, with the highest crystallinity observed at 1 wt % CF. The composites also exhibited narrower melting peaks than neat PLA, suggesting the formation of more uniform and/or more perfect crystals in the presence of the fillers.^53,54^

### 3.3 Morphological Analysis of Foams

The morphologies in Figure 6 clearly distinguish the roles of CF and PTFE during cell growth and stabilization. In PLA/CF5, the rigid, nondeformable carbon fibers act as stress concentrators within the thinning cell walls (Figure 6b). The mismatch in deformability between the fibers and the stretched PLA melt promotes local wall thinning and rupture, consistent with the irregular cellular structure observed at low magnification (Figure 6a). In contrast, PLA/PTFE1 exhibits a distinct reinforcement mechanism. During melt processing, PTFE particles undergo in situ fibrillation, forming fibrils that bridge adjacent cell walls (Figure 6d). This interconnected network codeforms with the PLA melt, distributes stress more uniformly, and delays local thinning and rupture. Consequently, PLA/PTFE1 exhibits a more regular and uniform cellular morphology (Figure 6c) despite its slightly larger average cell size than PLA/CF5.

**Figure 6:**
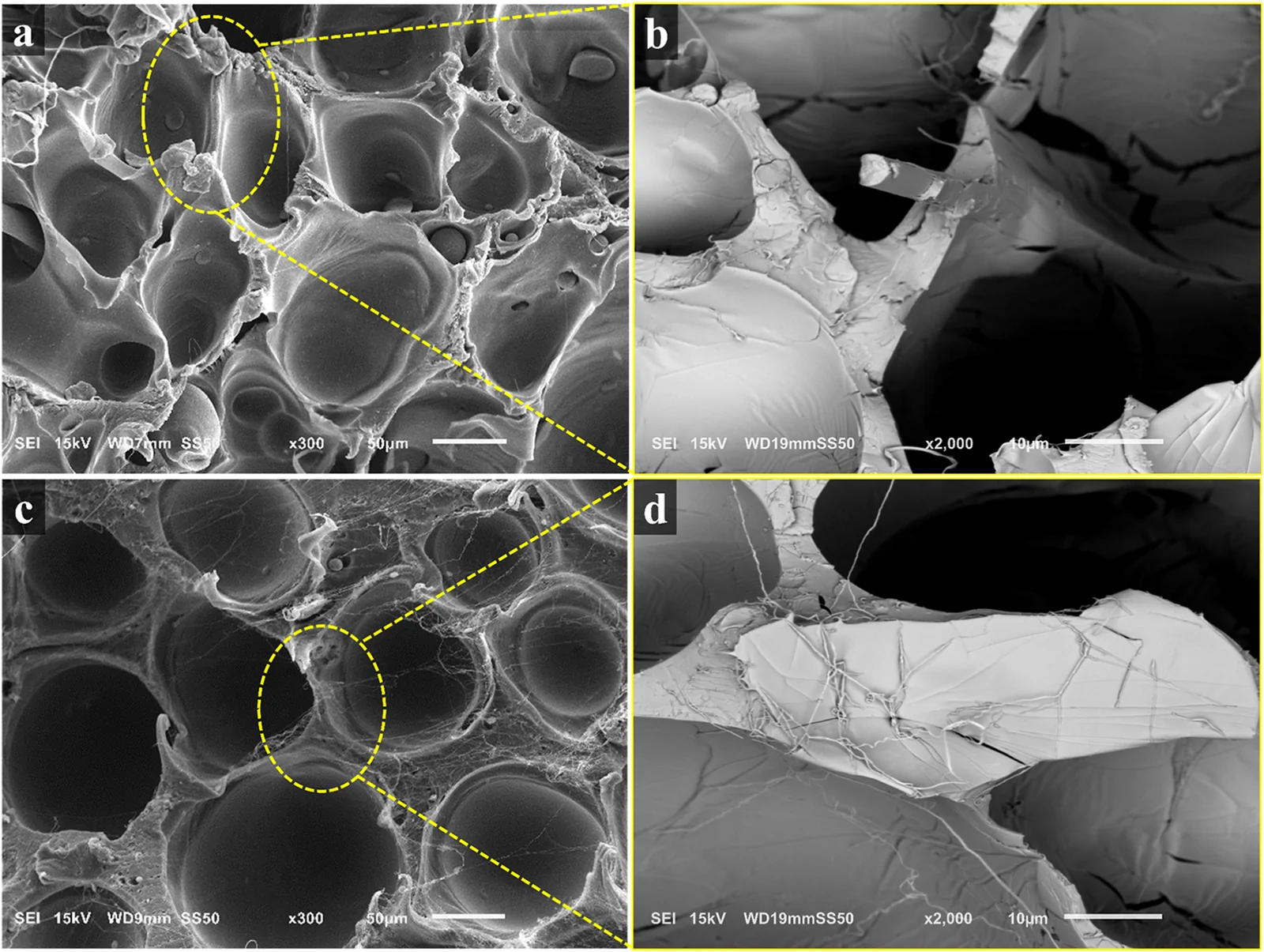
SEM micrographs of composite foams of PLA/CF5 at (a) 50 μm and (b) 10 μm and PLA/PTFE1 at (c) 50 μm and (d) 10 μm.

Figure 7 illustrates the foam morphology as observed in SEM of the pure PLA and PLA/PTFE/CF composite foams from the neck region, with the observation plane oriented perpendicular to the melt flow direction. In pure PLA foams, the cellular morphology is highly irregular, characterized by large, nonuniformly distributed cells that are elongated along the flow direction. This behavior is attributed to the inherently low melt strength of PLA, which promotes cell collapse and coalescence under shear during foaming.^55^ In contrast, the PLA/PTFE/CF composite foams exhibit significantly improved and more homogeneous cellular structures with smaller and more uniformly distributed cells. This improvement is associated with an enhanced melt strength and modified rheological behavior induced by the presence of PTFE/CF nanofibrils. These nanofibrils act as effective heterogeneous nucleating agents, promoting increased crystallization and generating a higher number of nucleation sites. The cell size distribution histograms clearly demonstrate the variation and distribution of cell sizes rather than relying solely on average values. For pure PLA (Figure 7a), a broad and skewed distribution is observed, with a higher fraction of large cells, confirming the nonuniform morphology and significant cell coalescence. In contrast, the PLA/PTFE/CF0.5 composite (Figure 7b) shows a narrower distribution with a higher frequency of smaller cell sizes, indicating more uniform nucleation and suppressed coalescence. With increasing filler content (Figure 7c–e), a gradual broadening of the distribution and a slight shift toward larger cell sizes can be observed.

**Figure 7:**
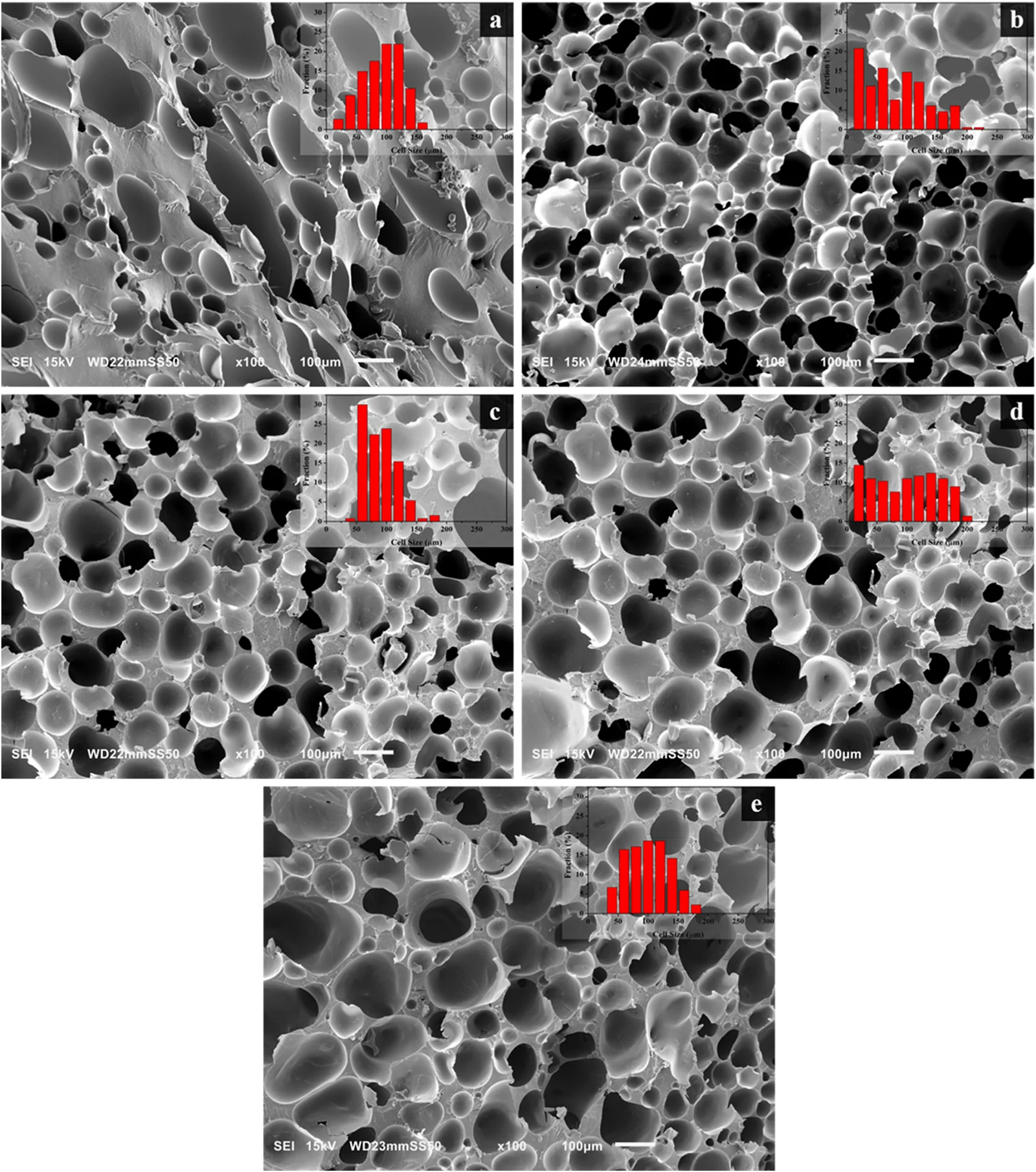
SEM images with cell size distribution histograms of foams: (a) pure PLA, (b) PLA/PTFE/CF0.5, (c) PLA/PTFE/CF1, (d) PLA/PTFE/CF3, and (e) PLA/PTFE/CF5.

The nature and concentration of nanofillers in the matrix play a significant role in the foaming process. The incorporation of 0.5 wt % CF resulted in a uniform cell structure, reducing the average pore size from 101.7 μm for pure PLA to 71.9 μm. A further increase in filler content beyond 1 wt % led to a gradual increase in cell size, indicating that the overall cell morphology of the composite foams remains largely unaffected. At higher filler contents, the average cell size moderately reached 90.7 μm at 5 wt % CF, while the composite foams continued to exhibit a well-developed cellular structure (Table S3).

Approximately more than 15% reduction in foam density was obtained for composites compared to pure PLA foams, showing that the interface area between CF and PTFE with PLA matrix may function as heterogeneous pore nucleation sites.^10^ All composites exhibited foam density in the range of 0.5–0.7 g/cm^3^, showing a balance between the number of cells formed and their volume expansion. The presence of fillers decreased the density of foams by more than 54% in reference to the compact samples (Figure 8a).

**Figure 8:**
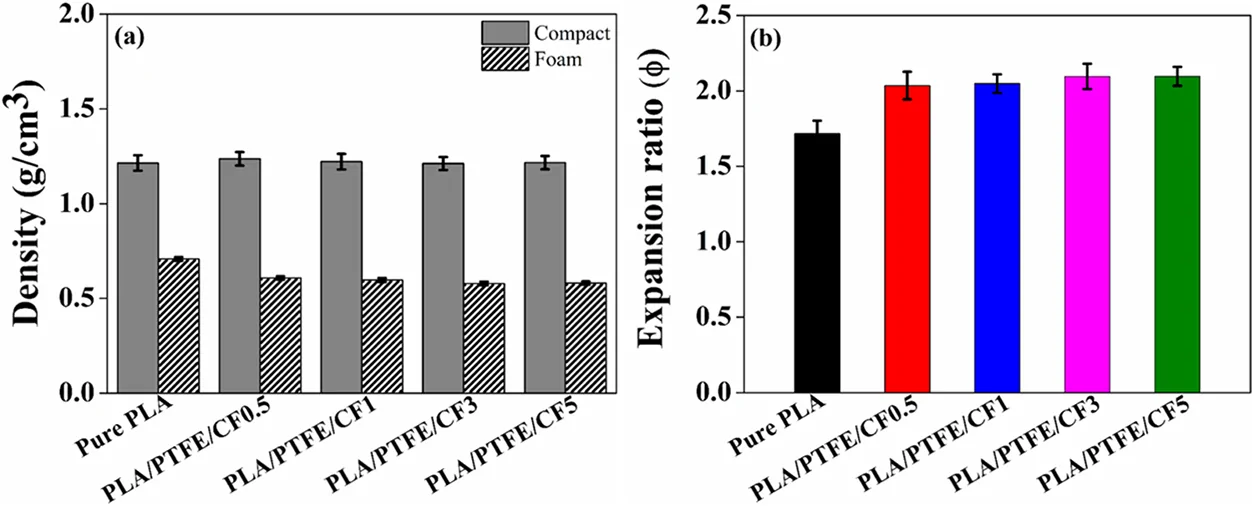
(a) Density of compact and foamed samples and (b) expansion ratio of foams.

With the introduction of fillers, the cell population density of the composite foams exhibited a significant increase from 1.55 × 10^6^ cells/cm^3^ for pure PLA to 4.16 × 10^6^ cells/cm^3^ for the PLA/PTFE/CF0.5 composite foam (Table S3). The addition of fillers enhanced the cell density for all composite foams, with values of 3.07 × 10^6^ cells/cm^3^, 2.71 × 10^6^ cells/cm^3^, and 2.41 × 10^6^ cells/cm^3^ for PLA/PTFE/CF1, PLA/PTFE/CF3, and PLA/PTFE/CF5, respectively, confirming the role of fillers in enhancing the cell density of PLA foams.

The X-ray tomographic analysis was performed to obtain the internal morphology of the molded samples non-destructively. Figure 9 shows the tomographic images of all samples captured from the neck region, which are consistent with the foam morphology observed by SEM in Figure 7. The pure PLA foams exhibit a large, nonuniform, and highly porous structure due to the low melt strength and viscosity of the materials during the foaming process. Upon incorporation of PTFE and CF, a notable change in the foam structure is observed. Both PLA/PTFE/CF0.5 and PLA/PTFE/CF1 samples show more uniform and reduced cell morphology, which can be attributed to the improved melt elasticity and the heterogeneous nucleation effect introduced by the addition of fillers that promote stable cell growth. At higher CF contents (3 and 5 wt %), partial cell coalescence and nonuniform cell structure were exhibited due to the excessive filler content.

**Figure 9:**
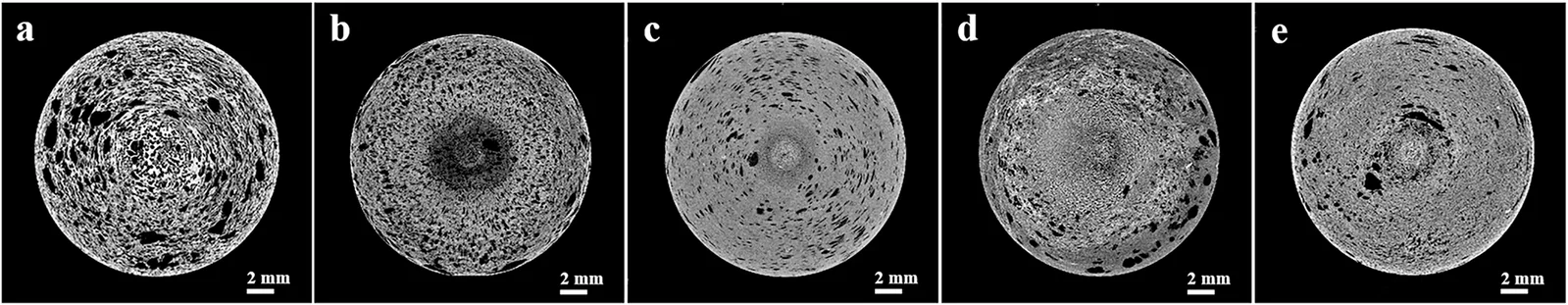
Tomographic images of injection-molded PLA foams: (a) pure PLA, (b) PLA/PTFE/CF0.5, (c) PLA/PTFE/CF1, (d) PLA/PTFE/CF3, and (e) PLA/PTFE/CF5.

The pore structure in three dimensions is best described using an equivalent spherical diameter, which allows for the comparison of irregular voids. The PLA/PTFE/CF0.5 structures exhibit small, relatively uniform pores with characteristic diameters in the range of 20–80 μm, suggesting controlled nucleation and limited coalescence. In contrast, the PLA/PTFE/CF1 structure shows a much broader distribution, with pores ranging from roughly 100 up to 500 μm or more, indicative of significant growth and merging during processing. PLA/PTFE/CF3 displays a bimodal distribution: small pores dominate the central region, while larger pores (100–600 μm) become increasingly prevalent toward the outer surface. In a defect-containing sample of PLA/PTFE/CF5, a fine background of 30–100 μm pores is interrupted by large macro voids reaching 0.5–1.5 mm, which are not part of the intrinsic foam structure but rather arise from gas entrapment or local phase separation. These observations align well with the average cell sizes reported in Table S3.

### 3.4 Compression Properties

To study the compression behavior of PLA/PTFE/CF foams, the neck portion of each sample was subjected to compression tests. The compressive stress–strain curves and compressive strength of pure PLA and the composite foams are presented in Figure 10.

**Figure 10:**
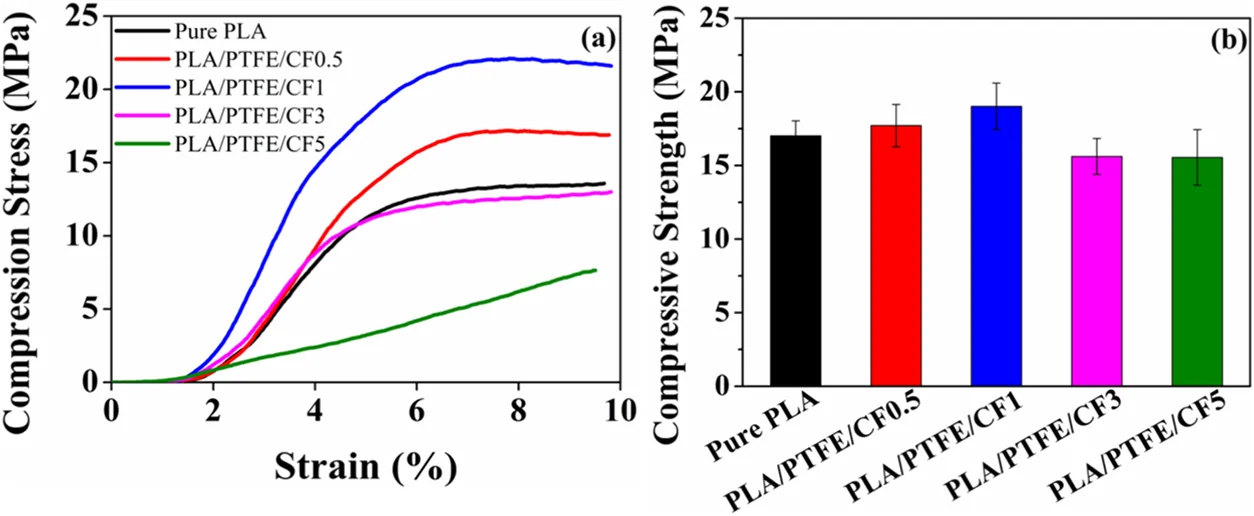
(a) Compressive stress–strain curves and (b) compressive strength of pure and composite PLA foams.

The linear elastic region in the curve (Figure 10a) corresponds to the elastic deformation of the cell walls, which determines the compressive strength and stiffness of the cell structure.^56^ With increasing deformation, the stress slightly increases with strain, corresponding to the gradual collapse of the cells as seen in the plateau region of the curve.^57^

As shown in Figure 10b, pure PLA foam exhibits lower compressive strength compared to PLA/PTFE/CF0.5 and PLA/PTFE/CF1 composite foams, which is associated with the large number of cell defects and weaker support possessed by the cell walls. The compressive strength of the composite foam increased with CF loading up to 1 wt %; specifically, the PLA/PTFE/CF1 composite foam demonstrated an 11.1% increment in compressive strength compared to pure PLA foam. This enhancement can be attributed to the introduction of PTFE and CF, which strengthened the extensional viscosity of PLA (Figure 4c), inhibiting cell coalescence and rupture. The presence of CF and PTFE fibrils provided heterogeneous nucleation sites, which helped in the formation of cells with reduced cell size (Table S3) and increased cell density, positively influencing the enhancement of the compressive strength of PLA foams. Similar mechanisms were observed for PMMA foams.^58^ Fillers acting as crystallization nucleating agents improved the crystallization properties of PLA, consequently enhancing the mechanical properties of the composite foams (Table S2). Furthermore, PTFE also served as an excellent reinforcing agent to improve the compressive strength of PLA foams due to its excellent physical properties.^59^ Compressive strength of PLA/CF/PTFE foams decreases beyond a CF concentration of 1 wt %, which is explained by an increase in average cell size with increased concentration of CF and by a significant decrease in PLA crystallinity, which is formed during foam solidification, as evidenced in Figure S4b. Thus, the cell size and cell morphology play a crucial synergetic role in determining the overall foam performance of foamed composites.

### 3.5 Thermal Insulation Properties

Figure 11 compares the thermal insulation performance of compact and foamed neat PLA and PLA/PTFE/CF composites. In both cases, filler addition improved thermal insulation relative to neat PLA. For the compact composites, thermal conductivity decreased from 0.20 W/m·K for neat PLA to 0.16 W/m·K at higher CF contents (3 and 5 wt %). Although CF has high intrinsic thermal conductivity along its longitudinal axis, its fragmented and randomly oriented distribution disrupts continuous heat-conduction pathways. In addition, the numerous fiber–matrix interfaces introduce thermal resistance, making the effective thermal conductivity strongly dependent on filler dispersion, orientation, and interfacial interactions.^60^

**Figure 11:**
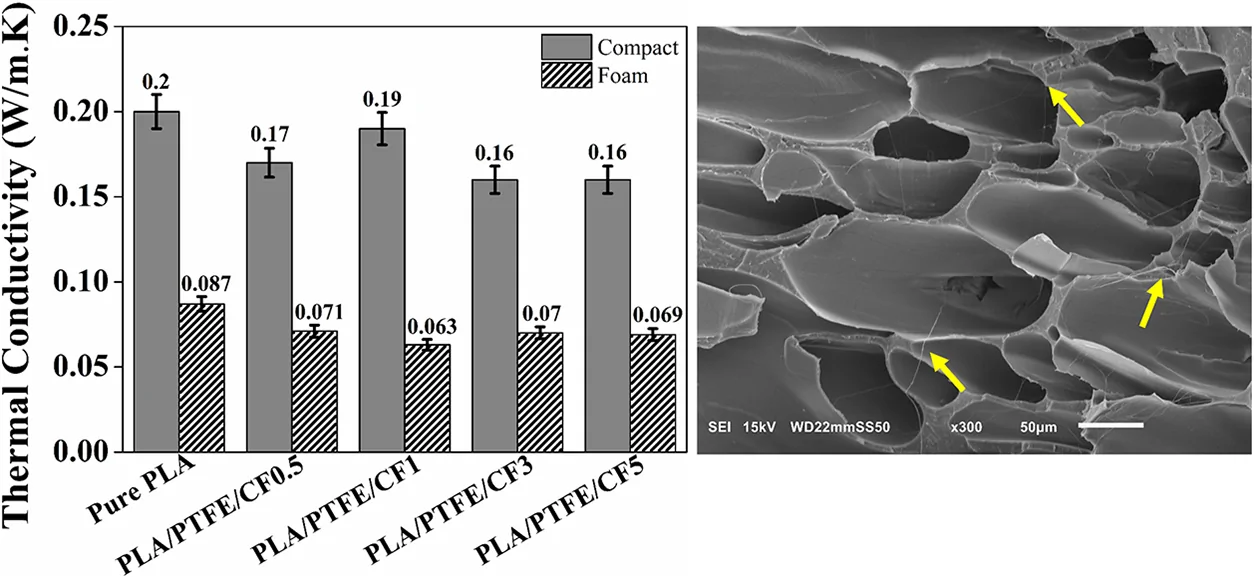
Thermal conductivity performance of compact and foamed samples.

The foamed samples exhibited a markedly lower thermal conductivity than their compact counterparts, confirming their superior insulation performance. This reduction is mainly attributed to the air trapped within the cellular structure, which has a low thermal conductivity. In polymer foams with cell sizes below approximately 4 mm, natural convection is negligible because the Rayleigh number of air remains below the critical value.^61^ Therefore, heat transfer is dominated primarily by conduction through the solid polymer phase and the gas phase.^62^ Among the foamed samples, PLA/PTFE/CF1 exhibited the lowest thermal conductivity and best insulation performance.

## 4 Conclusion

In this study, PLA-based nanocomposite foams were developed by incorporating in situ fibrillated PTFE and carbon fibers by using nitrogen-assisted foam injection molding. The influence of filler addition on the morphological, rheological, mechanical, thermal, and foaming behaviors was investigated. The composites were prepared by melt compounding, followed by foaming via an FIM technique. The morphological analysis revealed that the introduction of fillers significantly enhanced the strength of the matrix. The formation of the nanofibrillar PTFE network resulted in strong rheological reinforcement, improving the foamability of the composite. Furthermore, the improved melt viscosity induced by PTFE addition showed no CF agglomeration in all composites. The incorporation of fillers promoted heterogeneous nucleation, leading to uniform cell nucleation and a homogeneous foam structure. All composites exhibited foam densities in the range of 0.5–0.7 g/cm^3^, corresponding to approximately a 15% reduction in density compared with pure PLA foams, accompanied by a simultaneous increase in cell population density for all composite foams. X-ray tomography imaging further confirmed that the internal morphology of the samples was consistent with the observations from SEM foam morphological studies. The mechanical performance of the foams using compression tests showed that composite foams exhibited more than an 11% increase in compressive strength compared with pure PLA foam. The improvement was attributed to the synergistic effects provided by the interconnected PTFE/CF network, which enhanced the melt strength and distributed more efficient stress transfer throughout the matrix. Rheological analyses confirmed an increased storage modulus due to network formation between the polymer chains and nanofibrillar structures. Compounding of PLA with 1 wt % PTFE produced a network of PTFE nanofibers that substantially increased the viscous response of the melt, while the addition of only 0.5 wt % CF to the system drastically enhanced the elongational viscosity response, resulting in a strong strain-hardening behavior. The alliance of CF with the PTFE nanofibrillar network appeared to be effective through the formation of additional nodes in that network. The DSC results revealed a slight shift in the cold crystallization temperature toward a higher value with filler addition, indicating constrained molecular mobility and an enhanced nucleation effect within the composite. Additionally, all composite foams exhibited improved thermal insulation performance compared with pure foamed PLA. In summary, the developed PLA nanocomposite foam exhibited a promising strategy for lightweight structural and thermal insulation applications.

## Supplementary Material



## Notes

SEM micrograph of pristine CF and pure PLA; rheological (complex viscosity (η*), and (b) loss modulus) and thermal properties of composites; foam properties of injection molded foams; flexural properties of foams; and thermogravimetric Analysis (TGA) (PDF)
