# Supplementary material for: Nitrogen-Assisted Foam Injection Molding of Polylactide with Carbon Fibers and PTFE Nanofibrils
Source: Ind Eng Chem Res. 2026 Sep 17;65(38):20482–93. doi: 10.1021/acs.iecr.6c03418 (PMC13632784; doi:10.1021/acs.iecr.6c03418)
Supplement: Supplementary file 1 [file ie6c03418_si_001.pdf]

Supporting Information

Nitrogen-Assisted Foam Injection Molding of Polylactide with Carbon Fibers and PTFE Nanofibrils

Silla George Raju [1,2], Valentina Volpe [3], Rita Salomone [3], Hanieh Kargarzadeh [2], Roberto Pantani [3*], Andrzej Galeski [2*]

[1] BioMedChem Doctoral School of University of Lodz and Lodz Institute of the Polish Academy of Sciences, 21/23 Matejki Street, 90-237 Lodz, Poland

[2] Centre of Molecular and Macromolecular Studies, Polish Academy of Sciences, Sienkiewicza 112, 90-363 Lodz, Poland

[3] Department of Industrial Engineering, University of Salerno, Via Giovanni Paolo II 132, Fisciano 84084, Salerno, Italy

*Corresponding authors: andrzej.galeski@cbmm.lodz.pl , rpantani@unisa.it

Table S1. Processing parameters used in FIM process

**Processing Parameters**	**Value**
Temperature Profile (°C) [from hopper to nozzle]	175-180-185-190
Nozzle temperature (°C)	200
Filling pressure (hydraulic system) (bar)	110
Screw rotation speed (rpm)	150
Injection speed (%)	30
Dosage (mm)	15
Cooling time (s)	45
Counter Pressure (bar)	2

Morphological properties of composites

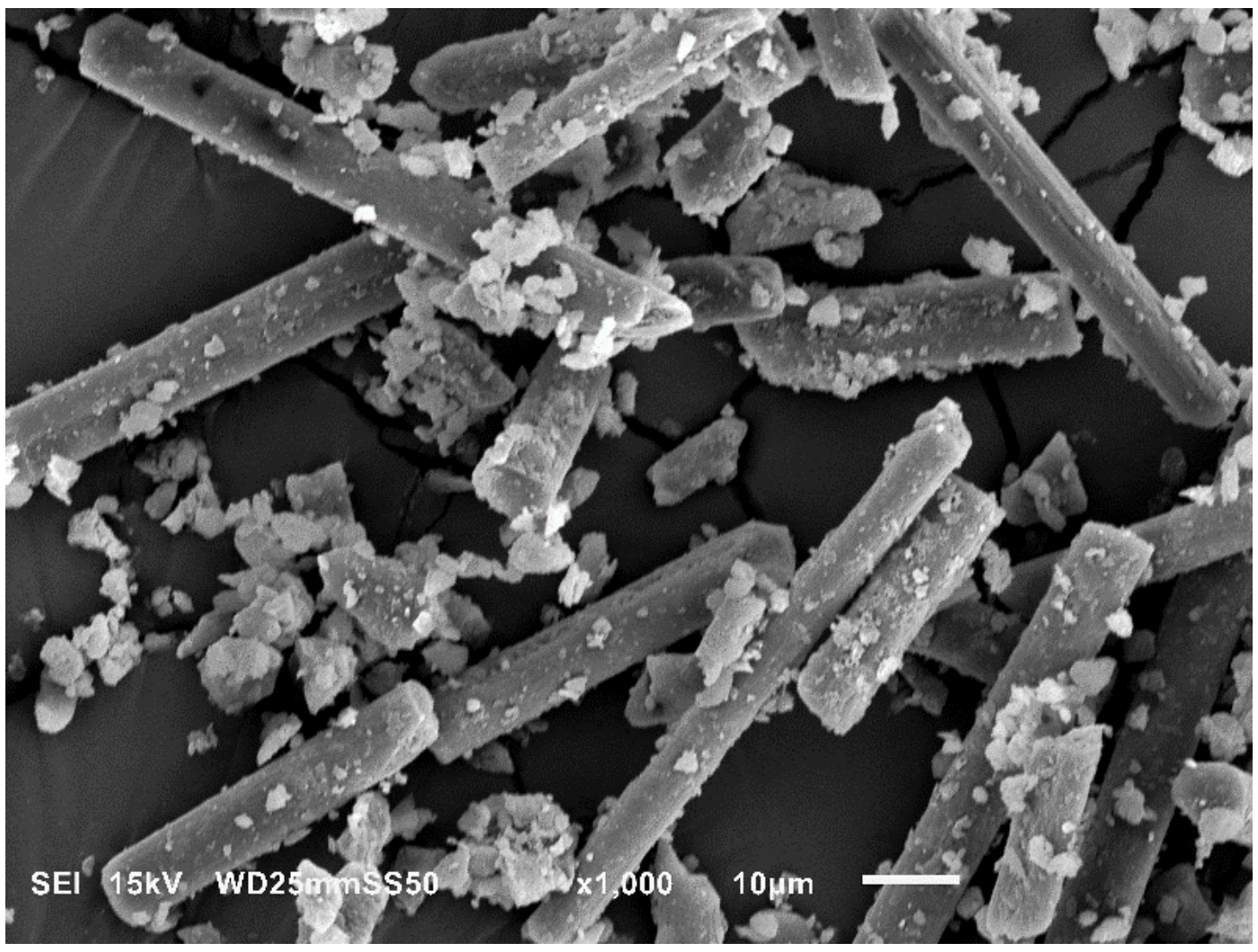

Figure S1. SEM micrograph of pristine CF.

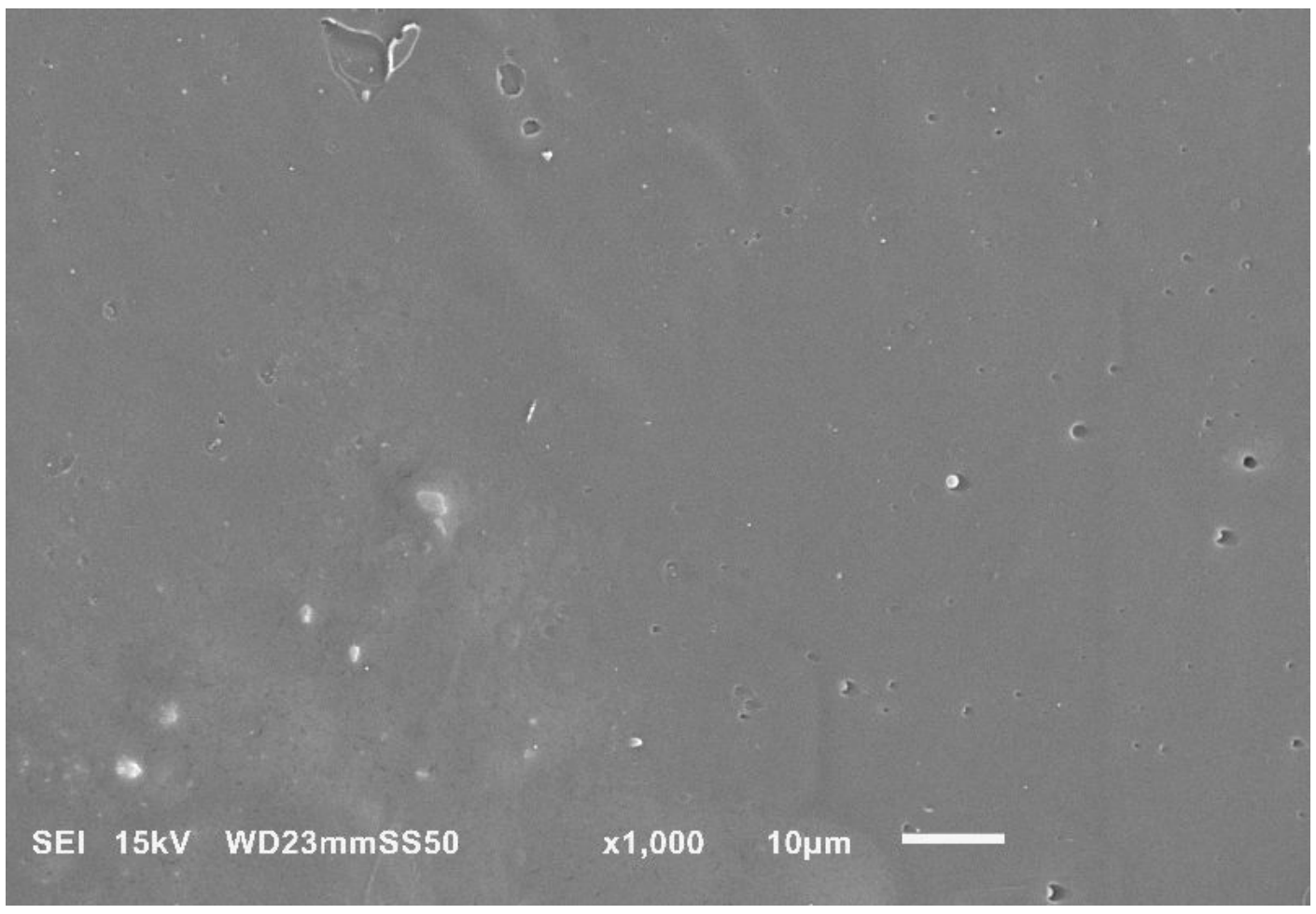

Figure S2. SEM micrograph of pure PLA.

Rheological properties of composite

Figure S3 shows the frequency dependence of the complex viscosity (η*) and loss modulus (G″) of neat PLA and its composites at 180 °C. At 0.5 wt% CF, η* decreased relative to neat PLA, possibly due to increased disruption of the PLA chain structure. At higher CF contents, η* increased, particularly at low frequencies, indicating the formation of interconnected PLA–PTFE–CF, CF–CF, PTFE–PTFE, and PTFE–CF networks. These networks restrict PLA chain mobility and increase resistance to flow. The higher viscosity at increased filler contents therefore reflects stronger polymer–filler and filler–filler interactions, promoted by the fine particle size and good dispersion of the fillers.

Frequency sweep measurements at different temperatures further showed that η* decreased with increasing temperature for both neat PLA and the composites. At lower temperatures, stronger polymer–filler interactions and a more interconnected filler network restrict chain mobility, resulting in higher viscosity.

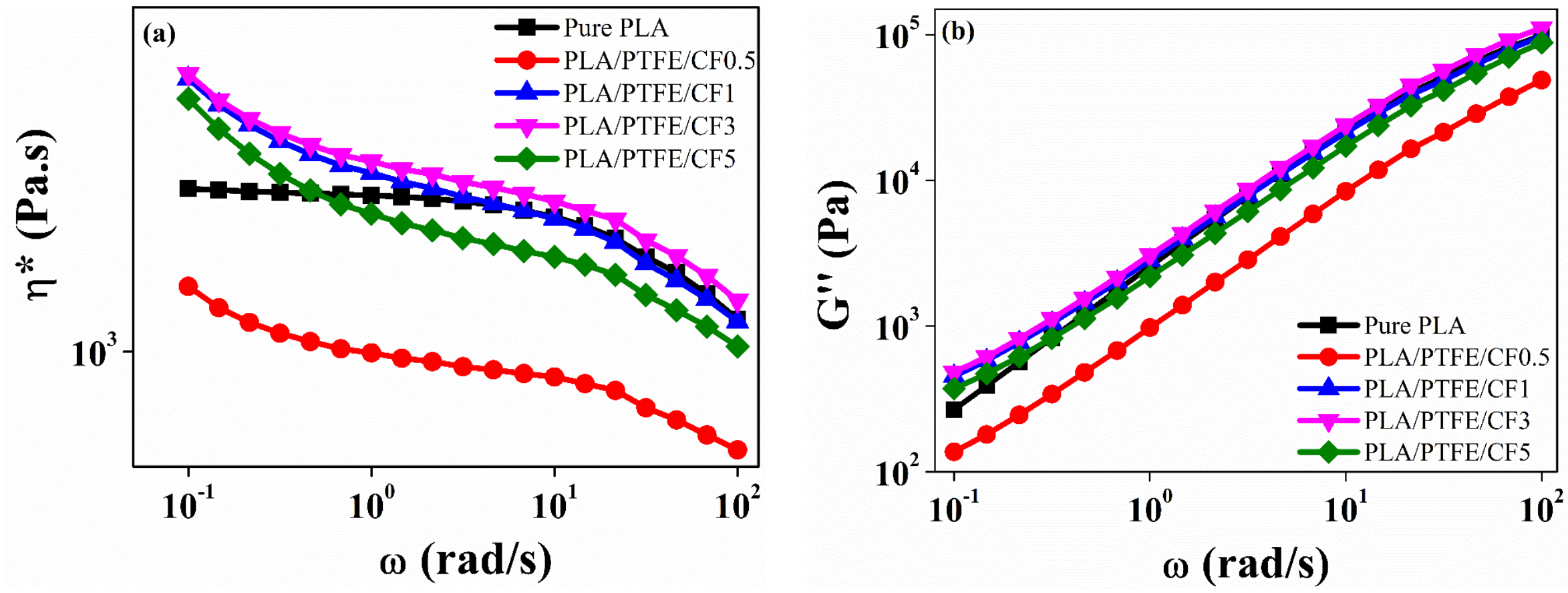

Figure S3. Rheological behaviour of pure PLA and its composites: (a) complex viscosity (η*), and (b) loss modulus (G″) as function of angular frequency.

Thermal properties of composites

The degree of crystallinity for the composites with 0.5, 1, 3, and 5 wt% of CF is shown in Figure S4b. Increasing the CF content from 0.5 to 1 wt% significantly increased the crystallinity from 0.8% to 5.4%, respectively. However, further CF addition reduced crystallinity, indicating that 1 wt% is the optimum CF content for promoting PLA crystallization. At higher filler contents, the formation of polymer-filler and filler-filler networks may restrict PLA chain mobility and hinder the chain rearrangements required for crystallization. The melting temperatures of neat PLA and the composites were similar, whereas filler addition increased the melting enthalpy, with the highest crystallinity observed at 1 wt% CF. The composites also exhibited narrower melting peaks than neat PLA, suggesting the formation of more uniform and/or more perfect crystals in the presence of the fillers [52, 53].

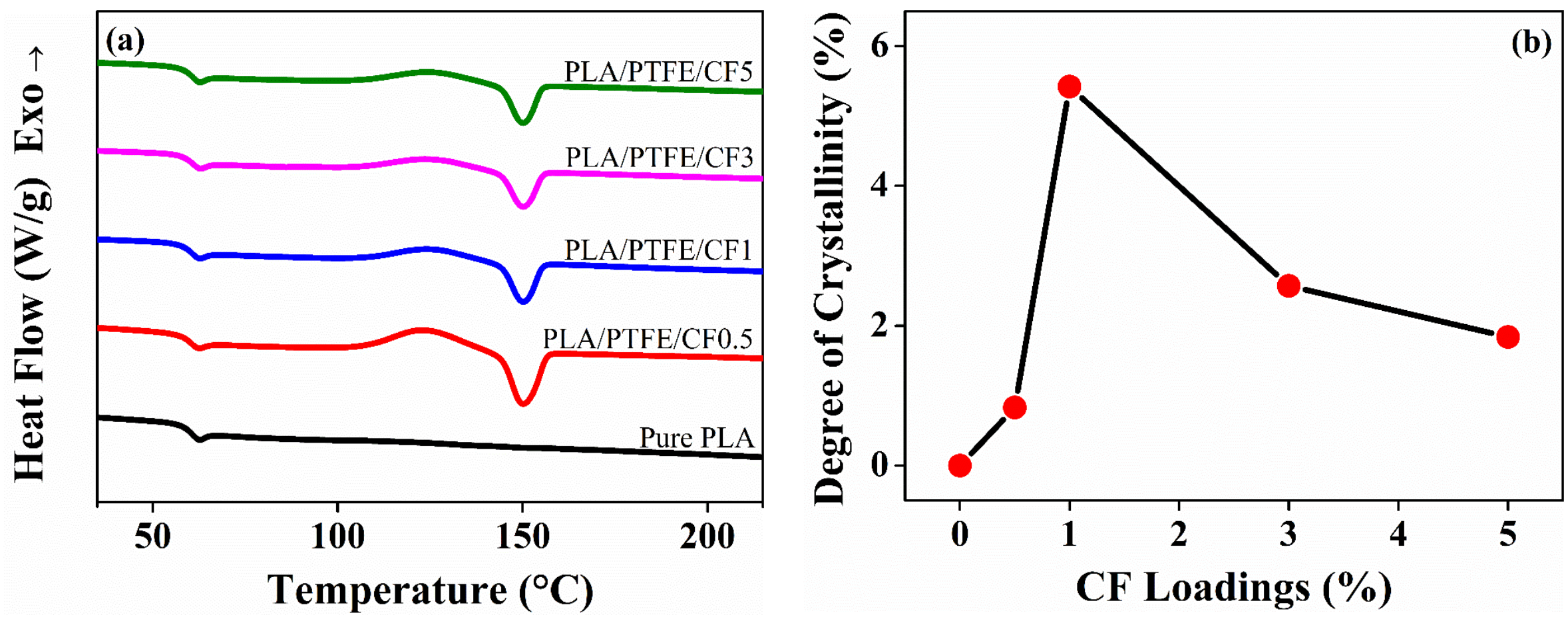

Figure S4. (a) DSC thermogram of second heating scan, (b) dependency of PLA degree of crystallinity in PLA/CF with CF loadings.

Table S2 Thermal parameters obtained from second-heating scan of DSC analyses on PLA samples.

**Sample**	T_g (°C)	T_{cc} (°C)	ΔH_c (J/g)	T_m (°C)	ΔH_m (J/g)	χ_c (%)
Pure PLA	59.5	-	-	-	-	-
PLA/PTFE/CF0.5	59.4	125.5	16.5	149.4	17.3	0.8
PLA/PTFE/CF1	59.4	126.1	7.5	149.3	12.4	5.4
PLA/PTFE/CF3	59.5	125.5	8.5	149.6	10.8	2.6
PLA/PTFE/CF5	59.5	123.8	9.4	149.1	11.1	1.8

Morphological analysis of foams

Table S3 Foam properties of injection molded foams

**Sample**	**Cell Population Density (cells/cm^3)**	**Average cell size (μm)**
Pure PLA	1.55×10^6	101.6
PLA/PTFE/CF0.5	4.16×10^6	71.9
PLA/PTFE/CF1	3.07×10^6	79.5
PLA/PTFE/CF3	2.71×10^6	87.8
PLA/PTFE/CF5	2.41×10^6	90.7

Flexural properties

The three-point flexural tests were carried out to evaluate the stiffness of foam specimens with the addition of PTFE/CF fillers. The tests were measured by means of universal testing machine ATSFAAR TC1000, with a load cell of 10 kN. The sample (40 mm diameter and 4 mm thickness) was placed on the testing apparatus, where the three-point support was regularly distributed on the base part of the sample. The load was applied at the center of the sample with a crosshead speed of 2 mm/min. The flexural behaviour of the specimens was analyzed from the base portion of the molded samples. The slope of each stress-strain curve, denoted as S_0, was used to determine the corrected slope parameter, S_P, according to the equation 1:

$$S_P = \frac{S_0}{0{\cdot}59\left(1-exp\left(\frac{-4{\cdot}1\times\Delta R}{D}\right)\right)+1} \tag{1}$$

where D is the diameter of the circumference where supports are located and ΔR represents the radial extension of the specimen outside the three-point bending supports. The corrected slope, S_P was subsequently normalized using the equation 2:

$$S_{PN} = \frac{S_P}{S_{P_0}} \cdot \frac{\rho_0}{\rho_f} \tag{2}$$

where S_{P_0} is the corrected slope of the compact specimen, while S_P corresponds to the corrected slope of the foamed specimen. The normalized slope (S_{PN}) of foamed PLA specimens is presented in Figure S5. In comparison to pure PLA foam, it is found that the incorporation of PTFE/CF fillers into the PLA matrix enhanced the flexural behaviour of the composite foams up to 1 wt% of carbon fiber loading. Around 20.5% and 3.9% increases in flexural stiffness were obtained for PLA/PTFE/CF0.5 and PLA/PTFE/CF1 composite foams, respectively, relative to pure PLA foam. This improvement in flexural property suggests enhanced interfacial adhesion between the fillers and the matrix. However, the lowest flexural behaviour was observed for composite foam containing 3 wt% carbon fiber, with values lower than those of pure PLA foam.

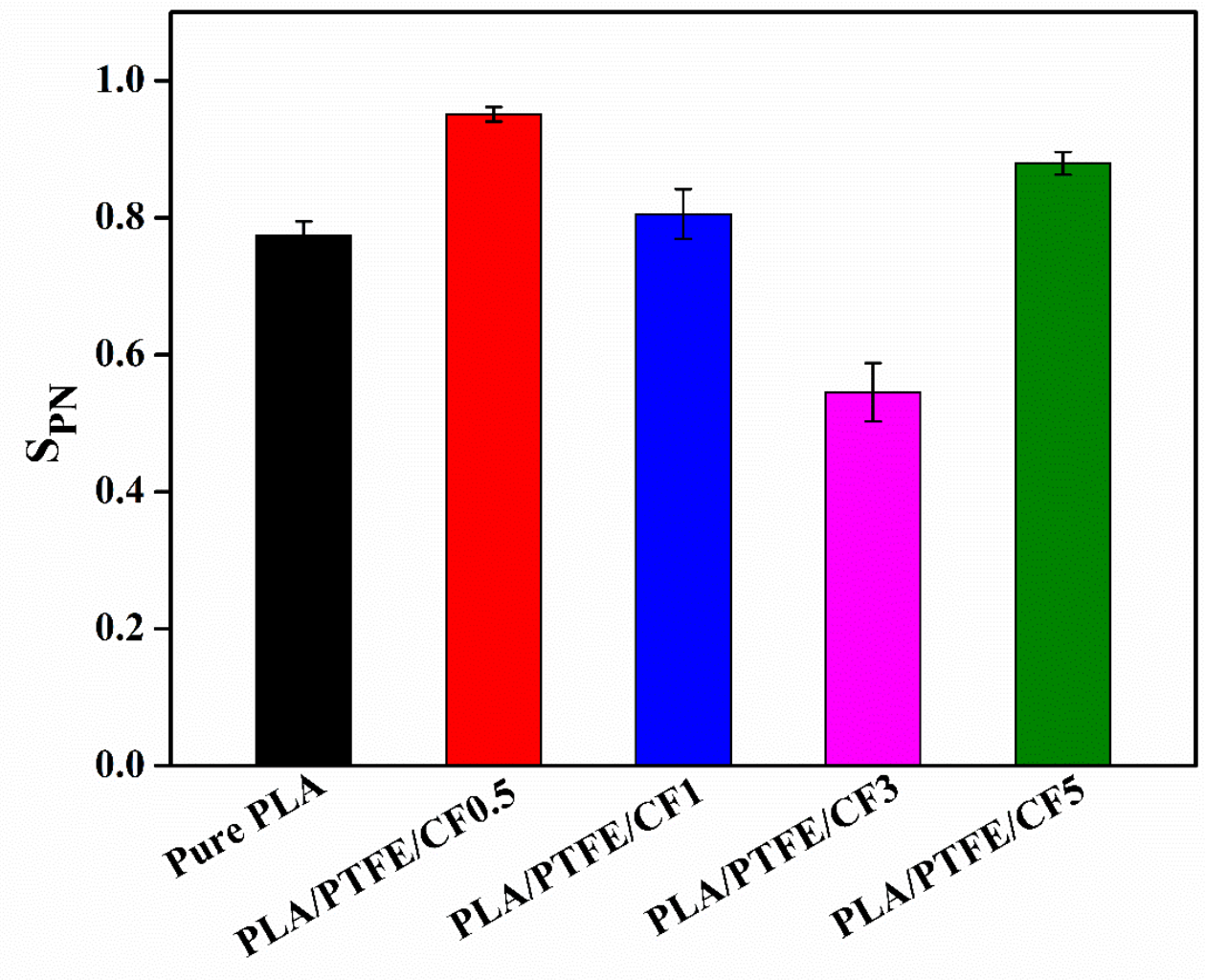

Figure S5. Normalized flexural slope, S_{PN}, of pure and composite PLA foams

Thermogravimetric Analysis (TGA)

Thermogravimetric analysis was conducted using a Mettler TC-10 (Mettler Toledo, Columbus, USA) thermobalance. The measurements were performed from 25 to 700 °C under an inert nitrogen atmosphere. At 700 °C, the purge gas was switched from nitrogen to air to continue the testing under an oxidizing atmosphere up to 1100 °C at 10 °C/min. The thermogravimetric analysis plot of pure PLA and PLA/PTFE/CF composites is shown in Figure S6. For pure PLA, the degradation is initiated at 352.9 °C, whereas composites containing 0.5, 1, 3, and 5 wt% of CF exhibit degradation onset at 344.3, 346.2, 345.4, and 347.7 °C, respectively. These results indicate a slight reduction in the onset of degradation temperature of the composite samples compared to pure PLA. Nevertheless, all composite specimens remained thermally stable approximately until 340 °C. The observed reduction in stability can be attributed to the decrement in degree of crystallinity in the composites, while the constraint of molecular chain mobility around fibers can also have an impact in this phenomenon.

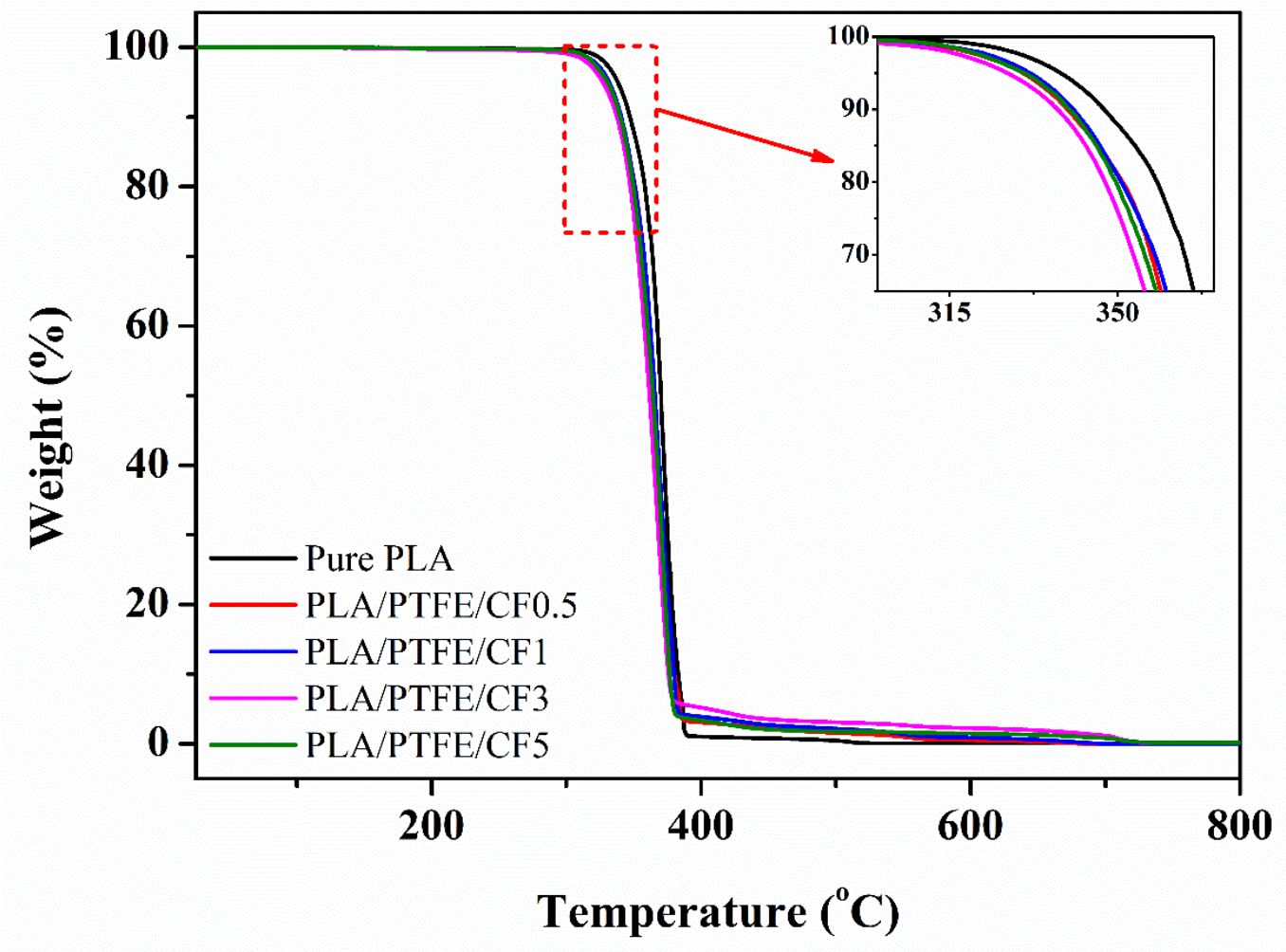

Figure S6. TGA graph of pure PLA and PLA/PTFE/CF composites
